# Current treatment status and barriers for patients with chronic HCV infection in mainland China: A national multicenter cross-sectional survey in 56 hospitals

**DOI:** 10.1097/MD.0000000000007885

**Published:** 2017-08-25

**Authors:** Dan-Dan Bian, Hai-Yang Zhou, Shuang Liu, Mei Liu, Carol Duan, Jin-Yan Zhang, Ying-Ying Jiang, Ting Wang, Yu Chen, Zhao Wang, Su-Jun Zheng, Zhong-Ping Duan

**Affiliations:** a Form Artificial Liver Center, Beijing YouAn Hospital, Capital Medical University; b Liver Department, Wu Jieping Medical Foundation, Beijing, China.

**Keywords:** DAAs, deferred treatment, HCV, patient satisfaction

## Abstract

Chronic hepatitis C virus (HCV) infection is a serious public health problem worldwide. China, as the country with the largest number of HCV infections in the world, plays a significant role in eliminating hepatitis C. Due to different financial situations and education background, hepatitis C patients take different actions for their disease treatment and management. Therefore, antiviral treatment status should be attached great importance to learn the medical demand of patients. A nationwide, multicenter survey was conducted from July 2015 to June 2016. Of 1798 inpatients and outpatients with chronic HCV from 56 hospitals participated in the survey. Each patient completed the questionnaire with questions about his/her antiviral therapy status, perception of treatment barriers, and expectations for future treatment. In total 1622 patients, including 1241 with chronic hepatitis C, 344 with cirrhosis, and 37 patients with hepatocellular carcinoma, fulfilled data collection requirements and finally were included in analysis. Overall, up to 30.7% of the patients had not or currently does not intend to receive antiviral therapy. The main reason was expecting more potent and well-tolerance medication (31.5%), followed by the fear of interferon related side effects (27.5%). Multiple regression analysis showed that the patient's annual income, the severity of HCV, and comorbidity were independent predictors of not receiving antiviral therapy. The whole patients were expecting more potent and well tolerance medication available soon. In summary, Peg-IFN/RBV treatment regimen cannot meet the need of patients well, and safe and efficient direct-acting antivirals are urgently needed in mainland China.

## Abstract 2

Supplemental Digital Content is available in the text

## 1 Introduction

Chronic hepatitis C virus infection is a major public health problem. Up to 185 million people worldwide are chronically infected with hepatitis C virus (HCV) and contribute to 350,000 deaths each year.^[1]^ Recent data estimated that at least 25 million individuals infected with HCV in China. And cases of HCV infection have been increasing steadily since 2003.^[2,3]^ The long-term HCV infection can lead to a series of liver disease from hepatic inflammation to extensive fibrosis and cirrhosis or hepatocellular carcinoma (HCC).^[2]^ Because the majority of HCV infection in China occurred in the late 1980s or early 1990s, with the progression of the disease, more and more patients develop into cirrhosis and hepatocellular carcinoma eventually. Therefore China is considered to have a huge burden of HCV associated cirrhosis and HCC.^[4]^

In recent years, dramatic improvements have been achieved in treatment of hepatitis C with the appearance and usage of direct antiviral agents (DAAs).^[5,6]^ Nevertheless, due to unavailability of DAAs in mainland China, Peg-interferon-α/ribavirin (Peg-IFN/RBV,P/R) is still the current standard treatment.^[7]^Although as early as 2004, China issued a “Guidelines of the management of hepatitis C” which has played a positive role for the promotion and application of standard treatment of hepatitis C, to the best of our knowledge, there is no report about the real status of HCV treatment in mainland China, especially from the viewpoint of CHC patients.

Chronic HCV infection is recognized as a global health problem, demanding an international, coordinated emphasis on prevention, management, and treatment.^[8]^The World Health Organization has recently initiated a global health strategy, which will run between 2016 and 2021, to eliminate hepatitis C as a global public health threat by 2030.^[9]^ China, as the country with the largest number of HCV infections in the world, plays a significant role in this event. In an effort to give data reference to the policy maker and health authority, we conducted this national, multicenter study to investigate the present status of HCV treatment and its influencing factors.

## 2 Methods

### 2.1 Study population

Fifty-six hospitals from all regions of mainland China (North, Northeast, East, Middle, South, and Northwest) participated in the study. Thirty-two of the 56 participating hospitals were general hospitals, 19 were hospitals specialized on infectious diseases, and 5 were traditional Chinese medicine (TCM) hospitals. The patients with a diagnosis of chronic HCV infection were eligible to participate in the study if they had tested serum positive for anti-HCV Ab or HCV-RNA at least 6 months before study enrollment, regardless of the age and sex.^[10]^ The diagnosis of “HCV related cirrhosis” was made on the basis of clinical, biochemical, ultrasonic, histological, radiological, and endoscopic findings and results, but should exclude the comorbidity of HCC. People who had been diagnosed with HCC, including those with and without a diagnosis of cirrhosis, were defined as “HCV related HCC.” The diagnosis of HCC was based on American Association for the Study of Liver Diseases guidance and radiology test results, alpha-fetoprotein serology, and/or biopsy.^[11]^ Patients were excluded from study participation if they had psychiatric disorders, could not cooperate with the investigation, or were unwilling to participate in the survey. Each subject signed the informed consent at the beginning of the study. The study protocol was conducted in accordance with the provisions of the Declaration of Helsinki and approved by the Institutional Review Board of Beijing YouAn Hospital, Capital Medical University (Beijing, China).

### 2.2 Questionnaire design and data collection

The questionnaire was finalized and examined by the expert committee. To ensure the stability and reliability of the questions, a pilot study was conducted before the comprehensive investigation. The stability of the questions was tested by answering the questionnaire by the same patients twice at an interval of 1 month and measured by calculating inter class correlation values, with an inter class correlation of greater than 0.8 indicating excellent stability. At the same time, we try to ensure that the respondents had enough time to fill in the questionnaire, so as to improve the reliability of the questionnaire.

To ensure the consistency of data collection, the principal investigators from all study medical institutions underwent training at Beijing YouAn Hospital before the study. The training curriculum included the interpretation of the inclusion and exclusion criteria, as well as introducing standard procedures for how to communicate with patients and how to guide them to complete the study questionnaires. Each site was asked to enroll 30 to 40 individuals, including at least 15 inpatients and 15 outpatients. Sites stopped enrolling new study participants when they reached this target. Investigation was conducted from July 2015 to June 2016. During this time, a clinical associate from Beijing YouAn Hospital was charged with responding to questions from the study sites.

### 2.3 Survey questions

This was a questionnaire survey. A 61-item questionnaire that consists of 4 parts was used for eligible respondents. The first section of the survey asked for the demographic and clinical characteristics of patients, such as age, sex, diagnosis, HCV genotype (GT), and so on. The second section included the questions on their current status of HCV treatment, including whether the patient received antiviral therapy, the names of antiviral medications and factors affecting the treatment status. The third section addressed the respondent's concerns and perception with anti-HCV treatment. Each respondent was presented with 17 potential barriers. Patients were asked to indicate their agreement to the statements. Each response was rated on a 10-point Likert scale, with 0 representing “not a barrier to treatment,” 5 representing “somewhat of a barrier to treatment,” and 10 representing “large barrier to treatment.” The fourth section collected the information of expectations about future treatment. Patients’ expectations about future treatment were assessed according to level of agreement with the following 8 statements: shorten period of treatment; convenient, no need for injection; improve therapeutic efficacy; reduce side effects, improve safety; reducing the frequency of monitoring; reduce the frequency of drug use. Each response was rated on a 10-point Likert scale, with 0 representing “strongly disagree,” 5 representing “neither agree nor disagree,” and 10 representing “strongly agree.” Persons were excluded from the analysis if they lose key data, such as the diagnosis of liver disease and treatment status. For HCV genotype and insurance information, we only analyzed the available data.

### 2.4 Data analysis

All data were analyzed using IBM SPSS Statistics for Windows, version 22.0 (IBM Corp, Armonk, NY). Continuous variables were described by average and standard deviation. Count material used frequency and rate. Independent *t* test, rank sum test, and *χ*^2^ test were used in comparison between 2 groups. Multivariate Logistic regression analysis was used to search for the influencing factors. A 2-sided *P* value of <.05 was considered statistically significant.

## 3 Results

### 3.1 Demographic and clinical characteristics of patients

**Figure 1:**
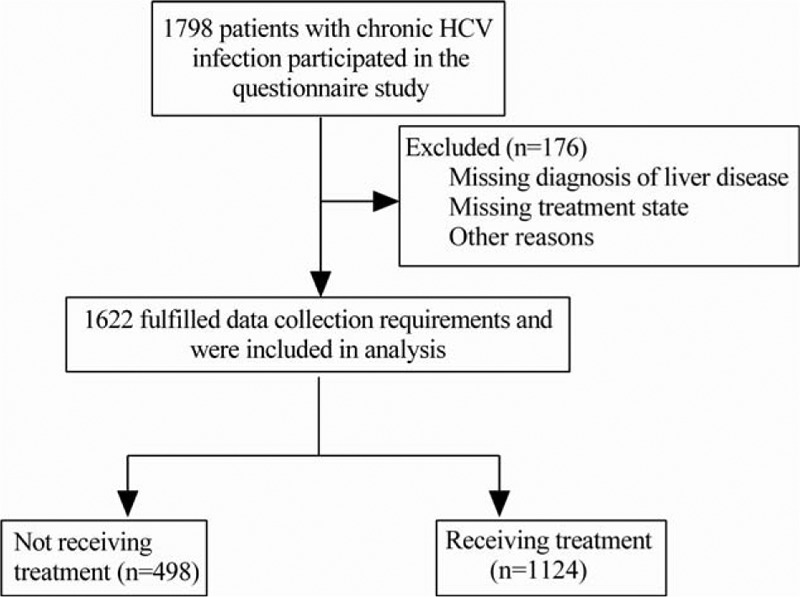
Flow of participants through this study.

Among 1798 questionnaires submitted, 176 persons were excluded from the analysis due to missing some key data, such as the diagnosis of liver disease. Of 1622 (90.2%) fulfilled data collection requirements and were included in analysis (Fig. 1). The variable distribution in the enrolled study population (n = 1622) is summarized as Table 1. Most patients were infected with HCV GT1 (34.9% [566/1622]) in patients with known genotypes and up to 45.9% (744/1622) of patients did not know their genotype. Sixty-five people had not given their information of medical insurance, so the remaining 1557 patients were included for further analysis. A greater proportion of patients (94.1% [1462/1557]) had medical insurance, most (89.9% [1400/1557]) were covered by the government public programs for urban and rural health, 6.1% (95/1557) of patients reported paying for medical expenses on their own. About 72.8% of the patients were older than 40 years old. Patients with chronic hepatitis C accounted for 76.5% (1241/1622), cirrhosis and HCC patients were 21.2% (344/1622) and 2.3% (37/1622), respectively (Table 1).

**Table 1:**
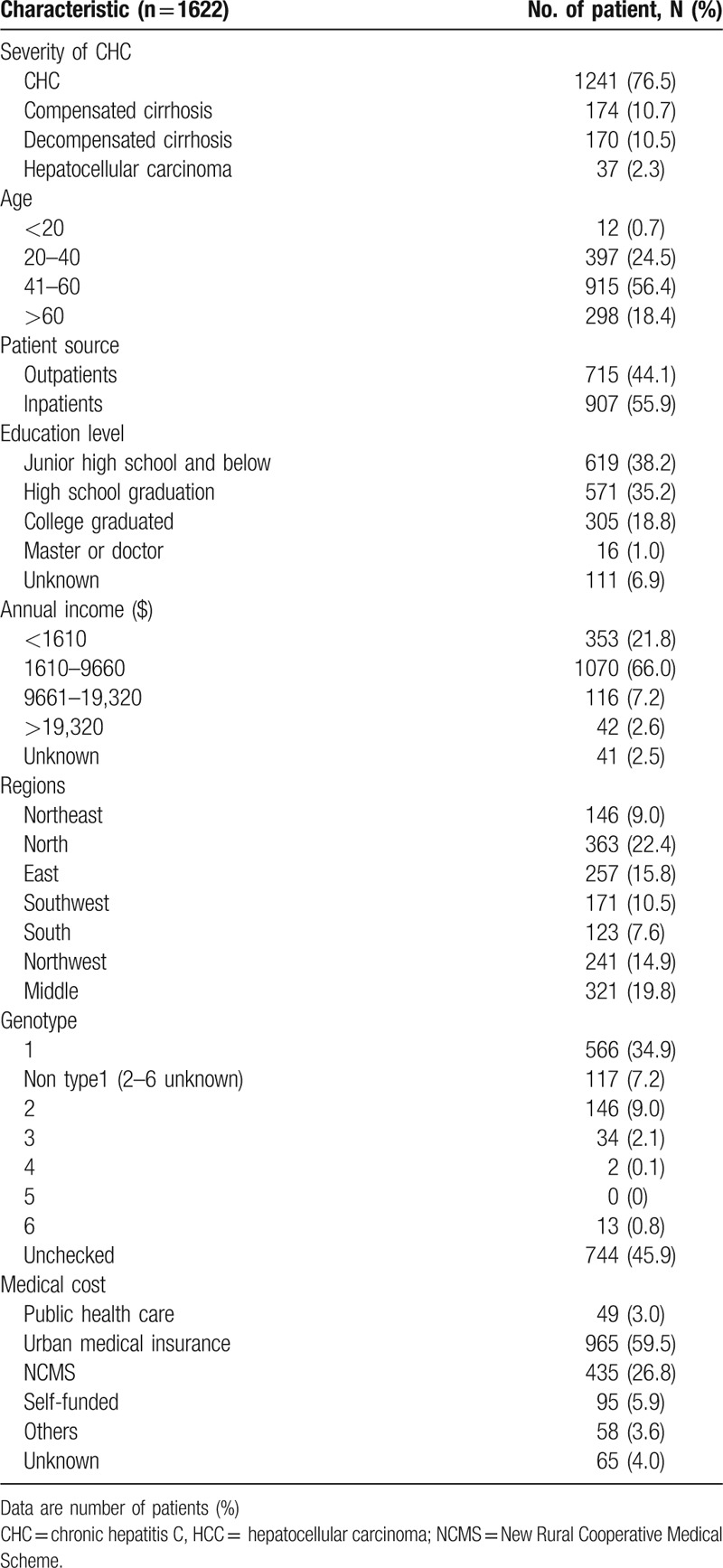
Demographic characteristics and clinical features of patients.

### 3.2 Treatment status and influencing factors

**Figure 2:**
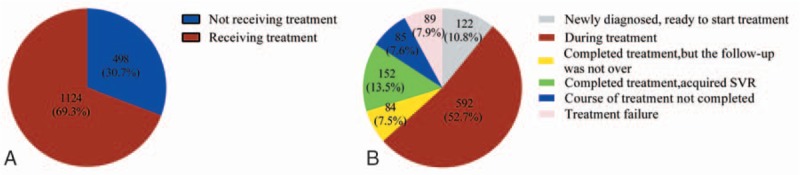
Treatment status of included chronic hepatitis C infection patients. A, Patients with chronic HCV infection receiving/not receiving P/R antiviral treatment. B, Antiviral status in chronic HCV infection patients. P/R = peg-interferon plus ribavirin (Peg-IFN/RBV), Not receiving treatment = not or does not currently intend to receive antiviral therapy, Receiving treatment = ready to treatment, in the course of treatment, treatment was not completed due to various reasons and treatment failure, HCV = hepatitis C virus.

Patients’ opinions regarding the antiviral therapy of HCV are shown in Fig. 2. Overall, for patients with CHC, up to 30.7% of the patients had not or currently did not intend to receive P/R antiviral therapy, and were classed as not receiving treatment group (Fig. 2A). Meanwhile, the receiving treatment group including the following several type of patients, 53% (1124/1622) in the course of treatment, 8% (85/1622) patients without completing treatment duration because of interferon or ribavirin intolerance or other reasons, another 8% (89/1622) patients with experienced treatment failure, 11% (122/1622) patients with new diagnosis and ready to treatment, 7% (84/1622) complete treatment, but has not yet reached the SVR24 when stop using the drug and 15% (152/1622) patients completed treatment, acquired SVR24 (Fig. 2B). A higher proportion of not receiving antiviral therapy patients had not medical insurance compared with receiving treatment patients (8.1% vs 5.2%, *P* = .029), Furthermore, the not receiving group had more severe liver diseases, elder age, lower-income and comorbidity compared with the receiving treatment group (Table 2). Comparison of antiviral therapy in different regions, we could find that the northwest region has a lower rate (56.0%) of antiviral treatment, the northeast region has a higher rate (75.3%) of antiviral treatment, but there was no statistical difference (*χ*^2^ = 12.6, *P* = .051) (Table S1).

**Table 2:**
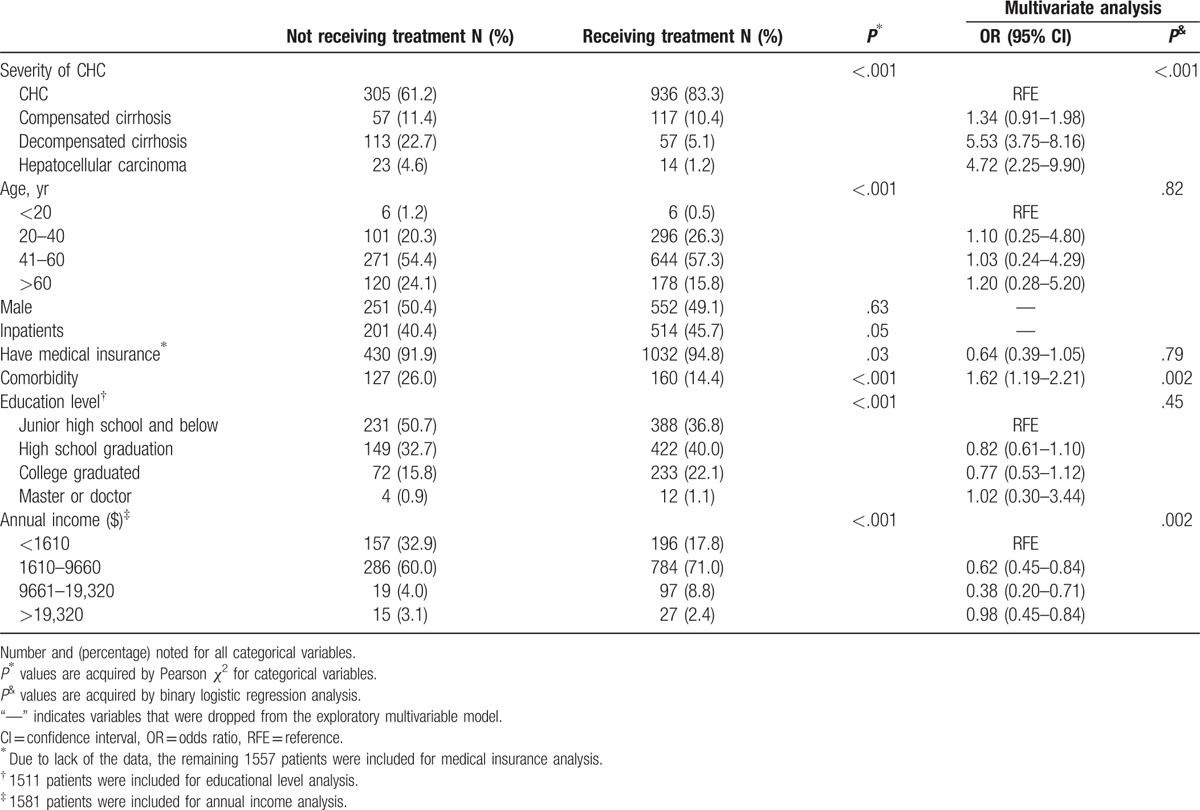
Differences in baseline demographics and disease characteristics between not and receiving treatment patients.

Multiple regression analysis showed that the patient's annual income (*P* < .001), the severity of CHC (*P* < .001), and comorbidity (*P* < .001), such as HBV, HIV, and kidney disease were independent predictors of the patients’ not receiving antiviral therapy (Table 2 and Table S2).

We further investigated the factors that influenced the choice of antiviral therapy between patients with different levels of income. Our results showed that in both low income (annual income <1610 dollars) and nonlow income patients (annual income> = 1610 dollars), the severity of CHC was all the independent risk factor. (Table S3, Table S4).

**Figure 3:**
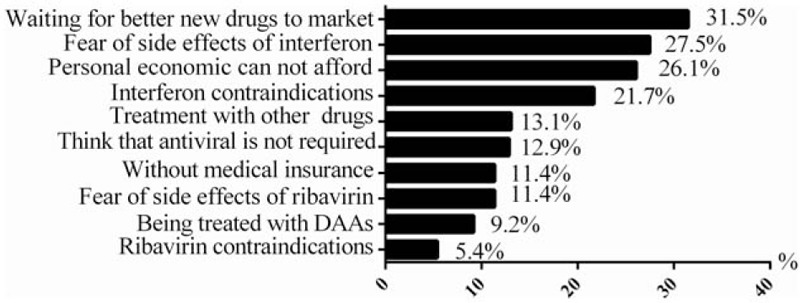
The reasons for patients had not or currently did not intend to receive P/R antiviral therapy. DAA = direct antiviral agent.

The reasons given by chronic HCV infection patients for selecting to defer P/R antiviral therapy were shown in Fig. 3. The most common cause given by nearly one-thirds of patients was waiting for a better new drug. Factors relating to the P/R treatment regimen itself were identified as a reason for not wanting to initiate antiviral therapy in many patients, and the leading reason was the fear of side effects of interferon (27.5% [137/498]), followed by existing contraindication or being intolerance to interferon (21.7% [108/498]). Approximately 26.1% (130/498) patients selected personal economic unaffordable as the main reason that they did not want to be treated.

### 3.3 Concerns, perception with anti-HCV treatment

Compared with patients receiving treatment, not receiving treatment patients had a higher cognitive impairment for anti-HCV treatment (Table 3). In particular, not receiving treatment patients had more obstacles than treatment group on “contraindications or intolerance to interferon (4.3 vs 5.8, *P* < .001) or ribavirin (4.0 vs 5.2, *P* < .001), and wanted to wait for new drugs to be marketed (3.8 vs 6.4, *P* < .001),” which presented with the receiving treatment group scores being less than 5 points, while no receiving group more than 5. We also found that despite the different degree, both group patients showed the fear of side-effect (6.2 vs 7.0, *P* < .001), and worrying the low success rate of P/R treatment (6.6 vs 7.2, *P* < .001) which presented with the scores being more than 5 points. Finally, in addition to treatment regimens, not receiving antiviral treatment patients still exist some other problems, such as poor recognition of HCV (5.4 vs 6.6, *P* < .001) and inadequate capacity to pay(5.3 vs 6.1, *P* < .001).

**Table 3:**
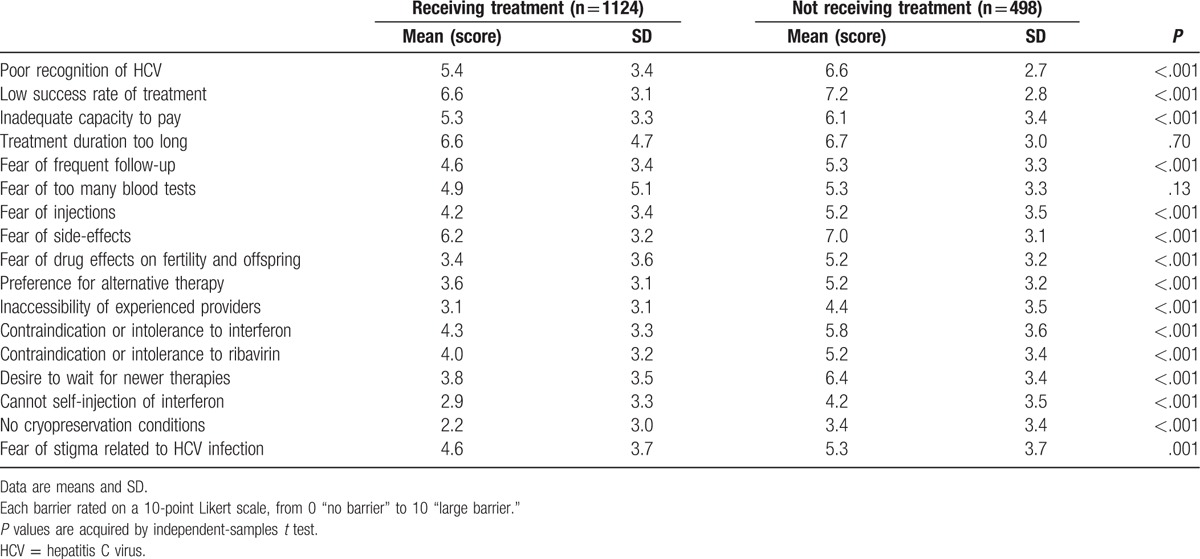
Differences in concerns, perception with anti-HCV treatment between not and receiving treatment patients.

As shown above, the patient's annual income was the independent predictors of patients’ not receiving antiviral therapy, we further analyzed the differences in “concerns, perception with anti-HCV treatment” between low and nonlow income patients (Table S5). We found that low-income patients had more obstacles than non-low income patients on “poor recognition of HCV (6.4 vs 5.6, *P* < .001)” and “fear of side-effects (6.8 vs 6.3, *P* = .012).” In addition, both group patients showed high obstacles on “worrying the low success rate of P/R treatment (7.0 vs 6.8, *P* *=* .222)” and “treatment duration too long” (7.0 vs 6.6, *P* *=* .040) which presented with the scores being more than 6 points.

### 3.4 Expectations about future treatment

Both groups of patients showed the similar strong expectations for new drugs with high efficacy, low side-effects and short duration in the future treatment (Table 4). Each expectation rated on a 10-point Likert scale, from 0 “not expecting” to 10 “very expecting.” Both groups showed expectations of up to 9 points or more in the 4 aspects: “improve therapeutic efficacy,” “shorten period of treatment,” “convenient, no need for no need for injection,” and “reduce side effects, improve safety.” All in all, the data indicated that the patients with HCV infection in mainland China are expecting more potent and well tolerance medication available soon.

**Table 4:**
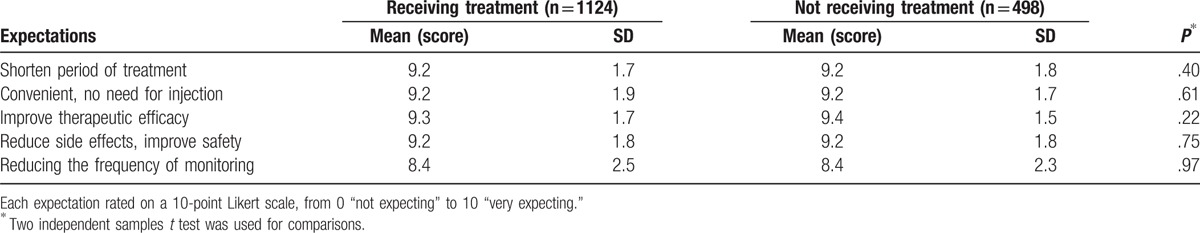
Expectations about future treatment.

## 4 Discussion

HCV is a curable disease now.^[12,13]^ Previously we have demonstrated that increasing the use of antiviral therapy for HCV in China can reduce the overall disease burden.^[4]^ Due to the high percentage of favorable host genotype IL-28B CC among Chinese patients,^[14]^ reported SVR rates of peg-IFN/RBV were relative higher than other ethnics.^[2]^ In spite of that, in present survey we first reported that a considerable proportion, nearly up to one third of CHC patients with chronic HCV infection declined currently available peg-IFN/RBV regimen. Not satisfied with peg-IFN/RBV treatment and expecting more potent and well tolerance medication were the major reasons. This finding will give data support for police maker of Chinese government.

In the survey, although fairly high proportions of chronic HCV infection patients reported being received antiviral treatment, 24.6% of chronic hepatitis C and 50.7% of HCV-associated cirrhosis and HCC reported receiving no treatment. Because of the aging of populations and delays in diagnosis and treatment of hepatitis C due to low public awareness of the disease, many Chinese patients seen in clinics are presented with advanced liver disease and loss the chance of P/R treatment.^[2]^ In this study, the proportion of patients with cirrhosis or HCC was as high as about 23.4%. As hepatitis C is a curable disease, earlier diagnosis and treatment would improve the outcome of HCV patients and will relief burden on the public health system in China. Therefore, it is necessary to strengthen publicity and education to improve the awareness of the importance of treatment for hepatitis C patients.

Comparison of baseline clinical and demographic characteristics between receiving treated and not receiving treated groups showed higher proportions of elder age, severe disease, and lower-income in not receiving antiviral therapy patients. This was consistent with previous studies in the United States and United Kingdom.^[8,15]^ It is easy to understand that patients with elder age and severe liver disease always are more intolerable to peg-IFN/RBV treatment. Notably, though, fairly high proportions of patients reported being covered by China's major types of government health insurance, health insurance was still affecting the patient's treatment options (91.9% vs 94.8%, *P* = .029). The finding deserves further attention from government policy-makers. Other research has shown that lack of health insurance for HCV patients will directly affect the health consequences. One US study reported that during the years 2005 to 2009, uninsured HCV patients in the United States had a 49% to 72% greater chance of dying during a hospitalization than HCV patients who had insurance.^[16]^ Apart from the inadequate coverage of health insurance, economic pressures also formed one of obstacles to the antiviral treatment. In our study, up to 32.9% of not receiving antiviral treatment patients have an annual income of less than $1610, while that of receiving treatment less than 17.8%. A study done in the United States also indicates that one-half of HCV patients cited personal financial resources as a barrier to care, despite 90% of patients possessing medical coverage.^[17]^

Comorbidity represents another significant barrier to HCV treatment. Factors such as kidney disease (*P* = .003), HBV (*P* = .015), and HIV (*P* = .025) all reduced the antiviral selection of HCV patients, except that diabetes mellitus (*P* = .105) had no effect on patient motivation. Combination with other diseases or coinfection increases the difficulty of treatment. Some diseases, such as severe renal damage, are not well fit for P/R antiviral therapy.^[18–20]^ While for coinfection with HBV, the increased risk of HBV DNA reactivation followed by antiviral HCV therapy should be concerned.^[21]^ Similarly, for HIV coinfection, the treatment regimen is often complex and brings more challenges to both patients and physicians.

Across all global regions, patient-level factors were viewed as the greatest obstacles to treatment.^[17,22–24]^ Specifically, fear of treatment-related side effects was the most frequently cited barrier.^[25,26]^ Consistent with this, our analysis of the reasons for patients not receiving P/R antiviral therapy indicated that the top 2 were “waiting for a better new drug” (31.5%) and “the fear of adverse effects” (27.5%). Considering the potential toxicity, low SVR acquirement, and long treatment duration of P/R treatment, it is easy to understand our data showed that the expected future treatment from both groups should have short course, high efficacy, easy to use, etc. DAA drugs almost meet all the above requirements, and have brought innovative revolutions to anti-HCV treatment. So, our data strongly suggested DAAs are urgent in need in current mainland China.

This study has some limitations. First, it is a cross-sectional investigation that does not reflect the dynamic changes of anti-HCV treatment. Second, the questionnaire was collected from hospital, which might have some deviation from the real world. Despite all this, the present 56 hospitals survey has provided strong evidences for both understanding the current anti-HCV treatment status and forecasting the huge demand of new anti-HCV treatment in mainland China.

## 5 Conclusions

To reduce the HCV public burden in China, early diagnosis of hepatitis C infection followed by more effective treatment is the key elements to combat HCV. There were many barriers that impede prompt and appropriate treatment of HCV infection in China. So, strengthening publicity and education, improving the patient's awareness of treatment, and improving medical insurance coverage are needed to achieve affordable and effective treatment of HCV in mainland China. Moreover, safe and efficient DAAs are urgently needed to be introduced into China to facilitate the global strategy of fighting against HCV infection.

## Supplementary Material

Supplemental Digital Content
