# Supplementary material for: Current treatment status and barriers for patients with chronic HCV infection in mainland China: A national multicenter cross-sectional survey in 56 hospitals
Source: Medicine (Baltimore). 2017 Aug 25;96(34):e7885. doi: 10.1097/MD.0000000000007885 (PMC5572026; doi:10.1097/MD.0000000000007885)
Supplement: Supplemental Digital Content [file medi-96-e7885-s001.doc]

| TABLE S1. Differences between not receiving treatment and receiving treatments from different regions. | | | | |
| --- | --- | --- | --- | --- |
| Regions | Not receiving treatment  N(%) | Receiving treatment  N(%) | χ^2^ | P* |
| Northeast | 36(24.7) | 110(75.3) | 12.6 | 0.05 |
| North | 97(26.7) | 266(73.3) |
| East | 67(26.1) | 190(73.9) |
| Southwest | 50(29.2) | 121(70.8) |
| South | 34(27.6) | 89(72.4) |
| Northwest | 106(44.0) | 135(56.0) |
| Middle | 108(33.6) | 213(66.4) |
| * Pearson chi-square test. | | | | |

| TABLE S2. Differences in co-morbidity between receiving and not receiving treatment patients. | | | | | | |
| --- | --- | --- | --- | --- | --- | --- |
|  |  | Not receiving treatment, N(%) |  | Receiving treatment, N(%) | χ^2^ | P* |
| Co-morbidity | Y | 58(19.5) |  | 105(11.4) | 12.7 | <0.001 |
|  | N | 240(80.5) |  | 817(88.6) |  |  |
| Kidney disease | Y | 15(5.0) |  | 17(1.8) | 9.0 | 0.003 |
|  | N | 283(95.0) |  | 904(98.2) |  |  |
| Diabetes | Y | 30(10.1) |  | 66(7.2) | 2.6 | 0.11 |
|  | N | 268(89.9) |  | 856(92.8) |  |  |
| HBV | Y | 15(5.0) |  | 21(2.3) | 6.0 | 0.02 |
|  | N | 283(95.0) |  | 901(97.7) |  |  |
| HIV | Y | 7(2.3) |  | 7(0.8) | 5.0 | 0.03 |
|  | N | 291(97.7) |  | 915(99.2) |  |  |
| * Pearson chi-square test; Y: Co-infection with other diseases, N: not co-infection with other diseases; HBV = Hepatitis B Virus, HIV = Human Immunodeficiency Virus. | | | | | | |

| TABLE S3. Differences in baseline demographics and disease characteristics between not receiving and receiving treatment patients with low income. | | | | | | | |
| --- | --- | --- | --- | --- | --- | --- | --- |
|  | | | Not Receiving treatment N(%) | Receiving treatment N(%) | P* | Multivariate analysis | |
|  |  |  |  |  |  | OR(95%CI) | P^&^ |
| Severity of CHC | | |  |  | <0.001 |  | <0.001 |
| CHC | | | 84(53.5) | 161(82.1) |  | RFE |  |
| Compensated cirrhosis | | | 20(12.7) | 21(10.7) |  | 1.58(0.77-3.25) |  |
| Decompensated cirrhosis | | | 47(29.9) | 14(7.1) |  | 5.80(2.82-11.93) |  |
| Hepatocellular carcinoma | | | 6(3.8) | 0(0) |  |  |  |
| Age（Years） | | |  |  | 0.31 | - |  |
| <20 | | | 6(3.8) | 5(2.6) |  |  |  |
| 20-40 | | | 24(15.3) | 44(22.4) |  |  |  |
| 41-60 | | | 88(56.1) | 107(54.6) |  |  |  |
| >60 | | | 39(24.8) | 40(20.4) |  |  |  |
| Male | | | 72(45.9) | 74(37.8) | 0.12 | - |  |
| Inpatients | | | 113(72.0) | 101(51.5) | <0.001 | 1.75(1.06-2.88) | 0.03 |
| Have medical insurance | | | 124(87.3) | 176(91.7) | 0.19 | - |  |
| [Co-morbidity](http://fanyi.baidu.com/#en/zh/co-morbidity) | | | 36(23.5)7 | 31(16.1) | 0.26 | - |  |
| Education level | | |  |  | 0.05 |  | 0.57 |
| Junior high school and below | | | 109(75.7) | 117(64.6) |  | RFE |  |
| High school graduation | | | 24(16.7) | 32(17.7) |  | 1.02(0.55-1.92) |  |
| College graduated | | | 10(6.9) | 29(16.0) |  | 0.57(0.25-1.29) |  |
| Master or doctor | | | 1(0.7) | 3(1.7) |  | 0.70(0.07-7.05) |  |
|  |  | Number and (percentage) noted for all categorical variables;  P* values are acquired by Pearson χ^2^ for categorical variables;  P^&^ values are acquired by binary logistic regression analysis;  OR=odds ratio, CI=confidence interval, RFE= reference;  “-” indicates variables that were dropped from the exploratory multivariable model; | | | | | |

| TABLES4. Differences in baseline demographics and disease characteristics between not receiving and receiving treatment patients withnon-low income. | | | | | | | |
| --- | --- | --- | --- | --- | --- | --- | --- |
|  | | | Not Receiving treatment N(%) | Receiving treatmentN(%) | P* | Multivariate analysis | |
|  |  |  |  |  |  | OR(95%CI) | P^&^ |
| Severity of CHC | | |  |  | <0.001 |  | <0.001 |
| CHC | | | 207(64.7) | 758(83.5) |  | RFE |  |
| Compensated cirrhosis | | | 36(11.3) | 94(10.4) |  | 1.29(0.84-1.98) |  |
| Decompensated cirrhosis | | | 60(18.8) | 43(4.7) |  | 4.42(2.83-6.89) |  |
| Hepatocellular carcinoma | | | 17(5.3) | 13(1.4) |  | 3.33(1.53-7.28) |  |
| Age（Years） | | |  |  | 0.002 |  | 0.34 |
| <20 | | | 0(0) | 1(0.1) |  | 1.00 |  |
| 20-40 | | | 72(22.5) | 251(27.6) |  | 0.84(0.55-1.28) |  |
| 41-60 | | | 172(53.8) | 525(57.8) |  | 0.72(0.51-1.03) |  |
| >60 | | | 76(23.8) | 131(14.4) |  | RFE |  |
| Male | | | 166(51.9) | 467(51.4) | 0.89 | - |  |
| Inpatients | | | 149(46.6) | 412(45.4) | 0.71 | - |  |
| Have medical insurance | | | 291(93.9) | 839(95.4) | 0.27 | - |  |
| [Co-morbidity](http://fanyi.baidu.com/#en/zh/co-morbidity) | | | 87(27.6) | 123(13.7) | <0.001 | 1.76(1.25-2.47) | 0.001 |
| Education level | | |  |  | 0.12 | - |  |
| Junior high school and below | | | 113(38.0) | 263(30.6) |  |  |  |
| High school graduation | | | 122(41.1) | 385(44.8) |  |  |  |
| College graduated | | | 59(19.9) | 203(23.6) |  |  |  |
| Master or doctor | | | 3(1.0) | 9(1.0) |  |  |  |
|  |  | Number and (percentage) noted for all categorical variables;  P* values are acquired by Pearson χ^2^ for categorical variables;  P^&^ values are acquired by binary logistic regression analysis;  OR=odds ratio, CI=confidence interval, RFE=reference;  “-” indicates variables that were dropped from the exploratory multivariable model; | | | | | |

| TABLE S5. Differences in concerns, perception with anti-HCV treatment between low and non-low income patients. | | | | | |
| --- | --- | --- | --- | --- | --- |
|  | Low income  N=(353) | | Non-low income  N=(1228) | | P |
|  | Mean (score) | SD | Mean (score) | SD |
| Poor recognition of HCV | 6.4 | 2.9 | 5.6 | 3.4 | <0.001 |
| Low success rate of treatment | 7.0 | 3.0 | 6.8 | 3.0 | 0.22 |
| Inadequate capacity to pay | 7.0 | 3.2 | 5.1 | 3.3 | <0.001 |
| Treatment duration too long | 7.0 | 3.1 | 6.6 | 4.6 | 0.04 |
| Fear of frequent follow-up | 4.7 | 3.4 | 4.8 | 3.3 | 0.62 |
| Fear of too many blood tests | 5.4 | 5.5 | 4.9 | 4.4 | 0.04 |
| Fear of injections | 4.8 | 3.6 | 4.4 | 3.4 | 0.04 |
| Fear of side-effects | 6.8 | 3.1 | 6.3 | 3.2 | 0.01 |
| Fear of drug effects on fertility and offspring | 4.3 | 4.0 | 3.5 | 3.7 | 0.002 |
| Preference for alternative therapy | 4.4 | 3.4 | 3.9 | 3.2 | 0.02 |
| Inaccessibility of experienced providers | 4.0 | 3.5 | 3.3 | 3.2 | 0.002 |
| Contraindication or intolerance to interferon | 4.9 | 3.5 | 4.6 | 3.4 | 0.25 |
| Contraindication or intolerance to ribavirin | 4.6 | 3.4 | 4.3 | 3.3 | 0.17 |
| Desire to wait for newer therapies | 4.3 | 3.6 | 4.6 | 3.7 | 0.13 |
| Cannot self-injection of interferon | 3.6 | 3.5 | 3.1 | 3.3 | 0.02 |
| No cryopreservation conditions | 2.9 | 3.3 | 2.4 | 3.1 | 0.004 |
| Fear of stigma related to HCV infection | 4.9 | 3.7 | 4.8 | 3.7 | 0.68 |
| Data are means and SD;  P values are acquired by independent-samples t test;  Low-income refers to the annual income of less than $1610 according to Chinese situation ;  Each barrier rated on a 10-point Likert scale, from 0 “no barrier” to 10 “large barrier.” | | | | | |
